# Data in support of proteomic analysis of pneumococcal pediatric clinical isolates to construct a protein array

**DOI:** 10.1016/j.dib.2016.01.057

**Published:** 2016-02-05

**Authors:** Alfonso Olaya-Abril, Ignacio Obando, Manuel J. Rodríguez-Ortega

**Affiliations:** a Departamento de Bioquímica y Biología Molecular, Universidad de Córdoba, Campus de Excelencia Internacional CeiA3, Córdoba, Spain; b Sección de Enfermedades Infecciosas Pe diátricas e Inmunopatología, Hospital Universitario Infantil Virgen del Rocío, Sevilla, Spain

**Keywords:** Pneumococcus, Protein arrays, Proteomics, Diagnostics

## Abstract

Surface proteins play key roles in the interaction between cells and their environment, and in pathogenic microorganisms they are the best targets for drug or vaccine discovery and/or development. In addition, surface proteins can be the basis for serodiagnostic tools aiming at developing more affordable techniques for early diagnosis of infection in patients. We carried out a proteomic analysis of a collection of pediatric clinical isolates of *Streptococcus pneumoniae*, an important human pathogen responsible for more than 1.5 million child deaths worldwide. For that, cultured live bacterial cells were “shaved” with trypsin, and the recovered peptides were analyzed by LC/MS/MS. We selected 95 proteins to be produced as recombinant polypeptides, and printed them on an array. We probed the protein array with a collection of patient sera to define serodiagnostic antigens. The mass spectrometry proteomics data correspond to those published in [1] and have been deposited to the ProteomeXchange Consortium [2] via the PRIDE partner repository [3] with the dataset identifier PXD001740. The protein array raw data are provided as supplemental material in this article.

## Specification table

**Table:** 

Subject area	Biology
More specific subject area	Microbial proteomics and immunology
Type of data	MS data, protein array data, tables
How data was acquired	Proteomic analysis of “shaved” bacteria was done using a Surveyor HPLC System in tandem with an LTQ-Orbitrap mass spectrometer (Thermo Fisher Scientific, San Jose, USA). Protein array data were acquired with a Genepix 4000B microarray scanner (Molecular Devices Corporation, Union City, CA)
Data format	Raw LC/MS/MS data; filtered and analyzed Excel files
Experimental factors	A collection of 24 invasive pneumococcal clinical isolates from pneumonia children patients was used; sera from two cohorts of children (patients with pneumococcal disease and patients with non-pneumococcal disease or healthy controls) were collected for subsequent protein array hybridization
Experimental features	The 24 pneumococcal clinical isolates were cultured in a chemically-defined medium and the live cells were trypsinized. The generated peptides were analyzed by LC/MS/MS, and a set of 95 proteins was chosen for recombinant polypeptide production and further protein array printing. The array was probed with a set of sera from children
Data source location	Córdoba and Sevilla, Spain
Data accessibility	Data are available at the ProteomeXchange: PXD001740 and also provided as supplemental material within this article. All of them are related to [1]

## Value of the data

• The “shaving” approach for identifying the “pan-surfome” of a collection of pneumococcal clinical isolates [4], [5] provides highly valuable information on the most promising surface antigens for vaccine and/or diagnostic purposes.
• The most promising protein candidates were produced as recombinant polypeptides to construct protein arrays.
• Sera from children patients were probed on the protein array to identify a set of immunodominant antigens that can be used for early diagnosis of infection.
• The strategy of combining experimental proteomics identification of surface proteins for protein array development proved to be very useful for its use in clinics with different utilities: diagnostics, epidemiological surveillance or vaccine discovery.

## 1 Data

Supplemental Dataset 1 contains all the raw protein array reads for patient and control sera, using both human anti-IgM and anti-IgG antibodies.

Table S1 shows the processed data (mean±SD) of the IgG Signal Mean Intensity (SMI) values of all sera used in the protein array hybridization measurements.

Table S2 shows the processed data (mean±SD) of the IgM Signal Mean Intensity (SMI) values of all sera used in the protein array hybridization measurements.

## 2 Experimental design, materials and methods

### 2.1 Experimental design

**Fig. 1:**
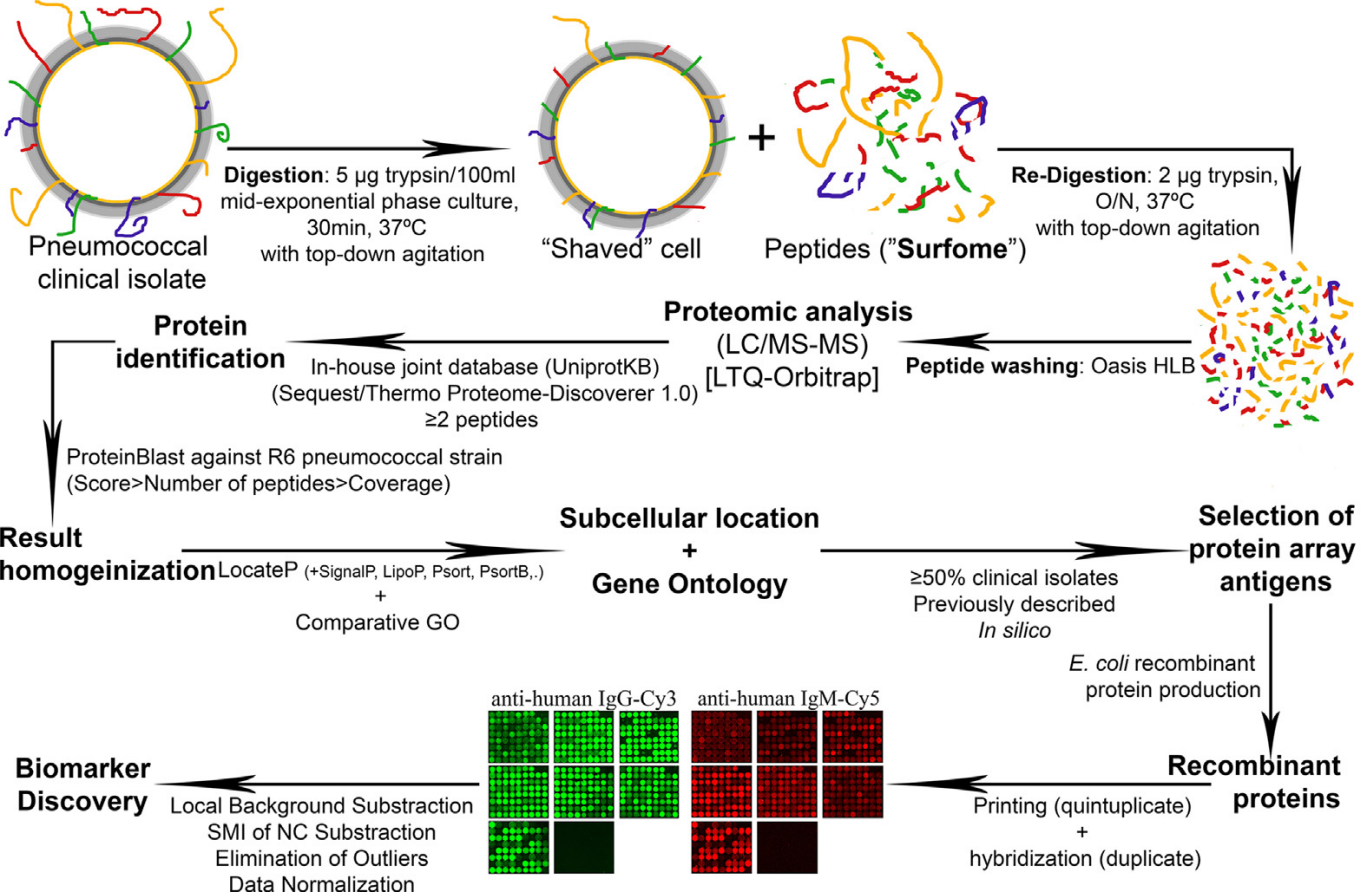
Flowchart of experimental design, data collection and processing.

A scheme for the experimental design from which the data were obtained is shown in Fig. 1. A collection of 24 clinical isolates from the Gram-positive human pathogen *Streptococcus pneumoniae*, also known as pneumococcus, were subjected to proteomic analysis to identify the set of the most prominent and abundant surface proteins (the “surfome”) in all or most of the analyzed isolates (the “pan-surfome”), following a strategy first described in [6], consisting of “shaving” the live cultured cells with trypsin. Then, the fractions of peptides generated from the surface-exposed and/or surface-attached proteins are redigested with trypsin as first described in [7] and optimized for pneumococcus in [4], to be analyzed by LC/MS/MS. The whole raw and processed data derived from those analyses, as further described in Section 3, can be found in the PRIDE repository with the dataset identifier PXD001740. The protein identifications were rearranged according to their subcellular localization and GO predictions, to select those predicted surface proteins and/or with assigned surface-linked functions present in a high proportion of clinical isolates. From this list, 95 proteins were finally selected for production of recombinant polypeptides according to the criteria already described in [1]. Also 9 predicted cytoplasmic proteins were selected, as they were identified very consistently and have been described to be surface-associated in numerous organisms [8]. Then, the 95 purified recombinant polypeptides were printed on a protein array and probed with a collection of human sera. After comparison of the “patient” and “control” groups (see below in Section 3), the potential serodiagnostic biomarkers were defined as those with at least a 1.5-fold difference in seroresponse between both groups.

## 3 Materials and methods

### 3.1 Human sera and ethical statement for their use

All human sera were obtained from patients admitted to Hospital Universitario Infantil Virgen del Rocío (HUIVR), Seville, Spain, and collected from children <14 years old. Sera were drawn either from patients with a diagnosis of pneumococcal pneumonia (the “patient” group), or from healthy children or patients affected by other pathologies different from pneumococcal pneumonia (the “control” group). Written informed consent was obtained from parents or legal guardians of participating children, for sera to be used within the project in which this work was designed, according to the principles expressed in the Declaration of Helsinki.

### 3.2 Bacterial culture and surface “shaving”

Twenty-four pneumococcal isolates from human patients with pleural empyema were kept, grown and “shaved” for surface protein identification as already described [4], [5].

### 3.3 LC/MS/MS analysis

All analyses were performed as described [4], [5], using a Surveyor HPLC System in tandem with an LTQ-Orbitrap mass spectrometer (Thermo Fisher Scientific, San Jose, USA) equipped with nanoelectrospray ionization interface (nESI). MS data (Full Scan) were acquired in the positive ion mode over the 400–1500 *m*/*z* range. MS/MS data were acquired in dependent scan mode, selecting automatically the five most intense ions for fragmentation, with dynamic exclusion set to on.

### 3.4 Database searching for protein identification

Tandem mass spectra were extracted using Thermo Proteome-Discoverer 1.0 (Thermo Fisher Scientific). Charge state deconvolution and deisotoping were not performed. All MS/MS samples were analyzed using Sequest (Thermo Fisher Scientific, version v.27), as described [1]. The raw data were searched against an in-house joint database containing 30,673 protein sequences from all the 17 full sequenced and annotated *S. pneumoniae* strains available at the UniProtKB site at the moment of the database construction (UniProt taxonomic IDs 189423, 488221, 574093, 561276, 516950, 373153, 487214, 488222, 488223, 171101, 487213, 525381, 760887, 512566, 170187, 1069625, and 760888, all of them in their versions of May 5, 2014).

### 3.5 In silico protein mining and functional annotation

Primary predictions of subcellular localization were assigned by using the web-based algorithm LocateP (http://www.cmbi.ru.nl/locatep-db/cgi-bin/locatepdb.py). They were contrasted by several feature-based algorithms: TMHMM 2.0 (http://www.cbs.dtu.dk/services/TMHMM-2.0) for searching transmembrane helices; SignalP 3.0 (http://www.cbs.dtu.dk/services/SinalP) for type-I signal peptides: those proteins containing only a cleavable type-I signal peptide as featured sequence were classed as secreted; LipoP (http://www.cbs.dtu.dk/services/LipoP) for identifying type-II signal peptides, which are characteristic of lipoproteins. GO annotations were retrieved from the UniProt Knowledgebase (http://www.uniprot.org/) and annoted using a webserver from the University of Adelaide (http://genomes.ersa.edu.au/BacteriaGO/submissions.php).

### 3.6 Production of recombinant proteins

Recombinant proteins were produced as double fusion fragments containing an *N*-terminal GST fragment and a *C*-terminal His-tag using the pSpark® I vector (Canvax Biotech, Córdoba, Spain), and expressed in *Escherichia coli* BL21, as described [1] and according to manufacturers’ instructions. Briefly, recombinant products were purified by Ni^2+^–agarose affinity chromatography from the *E. coli* intracellular fraction, dialyzed against PBS and used for protein array printing.

### 3.7 Protein array construction and probing

Affinity-purified recombinant proteins were printed on glass slides in quintuplicate (6 ng/spot) as detailed in [1] with split pins (4×4 pin tool) using a robotic array spotter (Genomic Solutions, BioRobotics MicroGrid II 610). Each component was prepared at 250 μg/ml in printing buffer (150 mM phosphate, pH 8.5, 0.01% sarkosyl) onto Nexterion Slide H 3-D glass slides. Eight complete arrays were printed on each slide. Probing with human sera was carried out in duplicate for each serum sample. Slides were assembled on 16-well slide holders (Nexterion Slide H MPX 16) and 45 µl of a dilution of different sera from the test set (1:200 in PBST) were incubated for 1 h protected from light at room temperature. The different samples were incubated with anti-human IgG-Cy3 (1:1000) or anti-human IgM-Cy5 (1:200), covered tightly with a seal strip, and incubated for 1 h at room temperature. To process the array data, the slides were scanned with a Genepix 4000B microarray scanner (Molecular Devices Corporation, Union City, CA) at photomultiplier voltage settings that no saturated pixels were obtained. Image analysis was carried out with Genepix Pro 4.1 analysis software (Molecular Devices Corporation). Local background subtraction was performed and corrected median feature intensity was used for initial data processing.

### 3.8 Protein array data analysis

For analysis of antibody binding to recombinant fragments on the microarray, local background subtraction from 10 surrounding spots was performed and corrected median fluorescence intensity was used for initial data processing. Then, the mean background signal of negative controls was substracted from each raw spot value after sera hybridization. Negative controls represented hybridizations of non-pneumococcal proteins and buffer spots with sera and secondary antibodies. Both in non-pneumococcal proteins and buffer positions, no reaction with human sera was observed. After background substraction, negative or zero values were assigned a net value of 0. Then, outlier values for each spot were removed. The two different hybridizations for each serum were averaged to report the signal mean intensity (SMI) values, and the mean and standard deviation (SD) were obtained from the 5 printed spots per protein in each patient and control groups. Finally, data normalization by background was carried out using Microsoft Excel as described [9].

## References

[1] Olaya-Abril A., Jimenez-Munguia I., Gomez-Gascon L., Obando I., Rodriguez-Ortega M.J. (2015). A pneumococcal protein array as a platform to discover serodiagnostic antigens against infection. Mol. Cell. Proteom..

[2] Vizcaino J.A., Cote R.G., Csordas A., Dianes J.A., Fabregat A., Foster J.M. (2013). The PRoteomics IDEntifications (PRIDE) database and associated tools: status in 2013. Nucleic Acids Res..

[3] Vizcaino J.A., Deutsch E.W., Wang R., Csordas A., Reisinger F., Rios D. (2014). ProteomeXchange provides globally coordinated proteomics data submission and dissemination. Nat. Biotechnol..

[4] Olaya-Abril A., Gomez-Gascon L., Jimenez-Munguia I., Obando I., Rodriguez-Ortega M.J. (2012). Another turn of the screw in shaving Gram-positive bacteria: optimization of proteomics surface protein identification in *Streptococcus pneumoniae*. J. Proteom..

[5] Olaya-Abril A., Jimenez-Munguia I., Gomez-Gascon L., Obando I., Rodriguez-Ortega M.J. (2013). Identification of potential new protein vaccine candidates through pan-surfomic analysis of pneumococcal clinical isolates from adults. PLoS One.

[6] Rodriguez-Ortega M.J., Norais N., Bensi G., Liberatori S., Capo S., Mora M. (2006). Characterization and identification of vaccine candidate proteins through analysis of the group A Streptococcus surface proteome. Nat. Biotechnol..

[7] Doro F., Liberatori S., Rodriguez-Ortega M.J., Rinaudo C.D., Rosini R., Mora M. (2009). Surfome analysis as a fast track to vaccine discovery: identification of a novel protective antigen for group B Streptococcus hyper-virulent strain COH1. Mol. Cell. Proteom..

[8] Olaya-Abril A., Jimenez-Munguia I., Gomez-Gascon L., Rodriguez-Ortega M.J. (2014). Surfomics: shaving live organisms for a fast proteomic identification of surface proteins. J. Proteom..

[9] Huber W., von Heydebreck A., Sultmann H., Poustka A., Vingron M. (2002). Variance stabilization applied to microarray data calibration and to the quantification of differential expression. Bioinformatics.

