# Supplementary material for: Data in support of proteomic analysis of pneumococcal pediatric clinical isolates to construct a protein array
Source: Data Brief. 2016 Feb 5;6:917–22. doi: 10.1016/j.dib.2016.01.057 (PMC4758182; doi:10.1016/j.dib.2016.01.057)
Supplement: Supplementary file 3 — Supplementary material [file mmc3.docx]

Dear Sirs from Data in Brief,

We declare no conflict of interests for the work submitted here.

Sincerely,

Prof. Manuel J. Rodríguez-Ortega, on behalf of the authors.
